# Enhanced Fusion Pore Expansion Mediated by the *Trans*-Acting Endodomain of the Reovirus FAST Proteins

**DOI:** 10.1371/journal.ppat.1000331

**Published:** 2009-03-06

**Authors:** Deniz Top, Chris Barry, Trina Racine, Chelsey Louise Ellis, Roy Duncan

**Affiliations:** Department of Microbiology & Immunology, Dalhousie University, Halifax, Nova Scotia, Canada; The Salk Institute for Biological Studies, United States of America

## Abstract

The reovirus fusion-associated small transmembrane (FAST) proteins are virus-encoded membrane fusion proteins that function as dedicated cell–cell fusogens. The topology of these small, single-pass membrane proteins orients the majority of the protein on the distal side of the membrane (i.e., inside the cell). We now show that ectopic expression of the endodomains of the p10, p14, and p15 FAST proteins enhances syncytiogenesis induced by the full-length FAST proteins, both homotypically and heterotypically. Results further indicate that the 68-residue cytoplasmic endodomain of the p14 FAST protein (1) is endogenously generated from full-length p14 protein expressed in virus-infected or transfected cells; (2) enhances syncytiogenesis subsequent to stable pore formation; (3) increases the syncytiogenic activity of heterologous fusion proteins, including the differentiation-dependent fusion of murine myoblasts; (4) exerts its enhancing activity from the cytosol, independent of direct interactions with either the fusogen or the membranes being fused; and (5) contains several regions with protein–protein interaction motifs that influence enhancing activity. We propose that the unique evolution of the FAST proteins as virus-encoded cellular fusogens has allowed them to generate a *trans*-acting, soluble endodomain peptide to harness a cellular pathway or process involved in the poorly understood process that facilitates the transition from microfusion pores to macrofusion and syncytiogenesis.

## Author Summary

The reovirus FAST proteins are the only known examples of nonenveloped virus membrane fusion proteins. Functioning as virus-encoded cellular fusogens, they mediate cell–cell membrane fusion and syncytium formation rather than virus–cell fusion. The FAST proteins are also the smallest protein fusogens and assume an unusual membrane topology, positioning the majority of their mass within or internal to the membrane in which they reside. We have been interested in reconciling the donor membrane-biased structural features of the FAST proteins with their ability to orchestrate the multi-step cell–cell membrane fusion process that leads to syncytium formation. We now show that the FAST proteins generate a soluble endodomain fragment that functions in *trans* from the cytosol, enhancing the capacity of diverse viral and cellular fusogens to drive the conversion of fusion pores into syncytia. The FAST proteins may therefore function in a similar manner as membrane receptors whose signalling activity requires regulated intramembrane proteolysis to generate a soluble signalling peptide. The endodomain signalling peptide of the FAST proteins provides a novel approach to identify cellular effectors involved in the fusion pore expansion stage of biological cell–cell membrane fusion.

## Introduction

The formation of multi-nucleated syncytia is an essential feature of a diverse range of biological processes [1]. Syncytiogenesis is contingent upon regulated cell–cell membrane fusion, which requires the involvement of protein catalysts to overcome the thermodynamic barriers that prevent spontaneous fusion of biological membranes [2]. The fusion proteins responsible for cell–cell fusion remain largely undiscovered and/or their mechanism of action poorly defined [1],[3]. Our current understanding of protein-mediated membrane fusion derives largely from the study of enveloped virus proteins designed to promote virus–cell fusion [4],[5], and from the SNARE proteins involved in intracellular vesicle fusion [6]. These studies converge on what may be a unifying model of membrane fusion involving a multi-step fusion-through-hemifusion pathway mediated by dynamic remodelling of the fusion protein complex [7],[8]. While mechanisms by which membrane fusion proteins promote membrane merger and the formation of focal fusion pores are beginning to emerge, relatively little is known about the processes that drive expansion of these fusion apertures, an essential step for those cell–cell fusion events that result in syncytium formation [9],[10].

The fusogenic orthoreoviruses encode a unique family of membrane fusion proteins, termed the fusion-associated small transmembrane (FAST) proteins. There are currently three distinct members of the FAST protein family named according to their molecular masses; p10, p14 and p15 [11]–[13]. Unlike enveloped virus fusion proteins, the FAST proteins are nonstructural proteins and are therefore not involved in promoting virus–cell fusion and virus entry [12],[13]. Following their expression inside virus-infected or transfected cells, the FAST proteins traffic to the plasma membrane where they perform their sole defined function, to induce cell–cell fusion and polykaryon formation in a wide variety of cell types [14]. The FAST proteins therefore function as promiscuous, virus-encoded “cellular” fusogens. The FAST proteins are both necessary and sufficient to induce membrane fusion, they need only be present in one of the two membranes being fused, and at only 95–140 residues in size, are the smallest known autonomous fusogens [15],[16]. All of the FAST proteins are single-pass membrane proteins that position very small N-terminal ectodomains (∼20–41 residues) external to the membrane and relatively larger C-terminal endodomains of ∼36–97 residues in the cytosol [11],[13],[17]. In contrast, most enveloped virus fusion proteins and the SNARE proteins are oriented with the majority of their mass positioned to interact with the proximal leaflets of the membranes to be fused [4],[6],[18]. We have been interested in reconciling the unusual topologies of the FAST proteins with their role as dedicated cell–cell fusogens.

Although enveloped virus fusion proteins can induce cell–cell membrane fusion, their primary function is to serve as virus–cell fusogens; their endodomains are therefore designed to function from the interior of the virion, not necessarily from the cytoplasm of the cell. This evolutionary imperative may explain why the endodomains of many enveloped virus fusion proteins either have no essential role in the membrane fusion reaction, or actually serve to inhibit cell–cell fusion activity, thereby coupling fusion competence to virion maturation [19]–[23]. In instances where the endodomain is required for membrane fusion, it is frequently involved in subcellular localization of the fusion protein, virus assembly and/or the formation of stable fusion pores [24]–[27]. As nonstructural viral proteins dedicated to executing cell–cell fusion, the endodomains of the FAST proteins do not need to inhibit fusion to promote virus assembly, and have specifically evolved to function during membrane fusion while in contact with the cytoplasm. These distinct evolutionary imperatives suggest the endodomains of the FAST proteins, and other yet to be identified cellular fusogens, might serve a different function during the fusion process than the endodomains of most enveloped virus fusion proteins.

The homologous p10 FAST proteins of avian reovirus (ARV) and Nelson Bay reovirus (NBV) contain 95–98 residues, distributed approximately equally on either side of the transmembrane domain [13]. The p14 FAST protein of reptilian reovirus is a 125-residue integral membrane protein, with a single transmembrane domain that separates a small, 36-residue N-terminal ectodomain from a considerably larger 68-residue C-terminal endodomain [11]. The asymmetric membrane topology of p14 is even more pronounced in the p15 FAST protein of baboon reovirus, which contains ecto- and endodomains of 20 and 97 residues, respectively [17]. Previous studies indicate that progressive deletion of the C-terminal endodomain of the p14 FAST protein leads to a progressive loss in cell–cell fusion activity, implying the C-terminal tail is essential for cell–cell membrane fusion [11]. The basis for this phenotype, however, has not been determined. We now show that ectopic expression of the FAST protein endodomains enhances the syncytiogenic activity of the full-length FAST proteins, both homotypically and heterotypically. Results further indicate that the biologically active endodomain fragment of the p14 FAST protein is endogenously generated from the full-length protein in virus-infected or transfected cells. Furthermore, the p14 endodomain peptide, when ectopically expressed in transfected cells, displays the surprising capacity to enhance syncytiogenesis mediated by unrelated viral or cellular fusogens. The syncytium-enhancing ability of the p14 endodomain is not dependent on interactions with either the fusogen or the membranes being fused, and occurs downstream of stable fusion pore formation. The FAST proteins are the first example of viral membrane fusion proteins that generate a soluble, bioactive endodomain fragment that presumably stimulates a cellular process central to the poorly understood sequence of events that promote the transition of stable fusion pores into syncytia.

## Results

### The p14 Endodomain Functions as a General Enhancer of Syncytiogenesis

**Figure 1:**
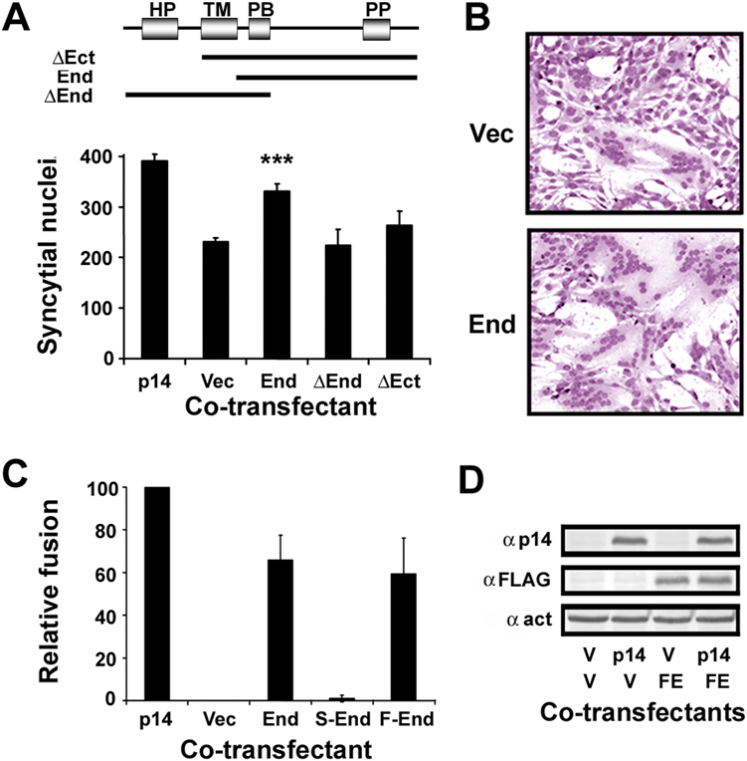
The p14 endodomain peptide enhances p14-induced syncytiogenesis. (A) Top panel: Arrangement of structural motifs in the p14 FAST protein, and the regions present in the N- (ΔEct) and C- (ΔEnd) terminally truncated p14 constructs and the endodomain construct (End) are depicted. HP, hydrophobic patch; TM, transmembrane domain; PB, polybasic region; PP, polyproline region. Bottom panel: QM5 fibroblasts transfected with a plasmid expressing p14 were co-transfected with plasmids expressing full-length p14 (p14), empty vector (Vec), the p14 endodomain (End), or p14 with a deleted endodomain (ΔEnd) or deleted ectodomain (ΔEct), and triplicate samples were quantified for the extent of cell–cell fusion at 8 h post-transfection. Values are the average number of syncytial nuclei per field±S.E. (n = 4). Only the endodomain construct, indicated with the asterisks, resulted in a statistically significant increase in syncytium formation (p<0.001) relative to the cells co-transfected with p14 plus empty vector. (B) The extent of syncytium formation present in cells co-transfected with p14 plus empty vector (Vec) or p14 plus the p14 endodomain (End) was visualized by bright field microscopy of Giemsa-stained monolayers at 8 h post-transfection. (C) Cells transfected with the p14 expression plasmid were co-transfected with plasmids expressing authentic p14 (p14), empty vector (Vec), the p14 endodomain (End), or a scrambled (S-End) or N-terminally FLAG-tagged (F-End) version of the p14 endodomain. The extent of cell–cell fusion was quantified as described in (A), and results are presented as the relative level of syncytium formation±S.E. (n = 4), setting the cells co-transfected with authentic p14 as 100% fusion enhancement and those co-transfected with empty vector as 0% fusion enhancement. (D) Cell lysates from cells co-transfected with the indicated expression plasmids (p14, authentic p14; V, empty vector; FE, FLAG-tagged p14 endodomain) at 12 h post-transfection were processed for Western blotting using antibodies against p14, the FLAG epitope, or β-actin (indicated on the left).

While analyzing a series of N-terminal truncations of the p14 FAST protein, we made the surprising discovery that co-expression of the p14 endodomain fragment (that induces no syncytium formation on its own) with the full-length p14 protein increased syncytiogenesis. Cells co-transfected with full-length p14 plus the p14 endodomain significantly increased the extent of syncytium formation relative to cells co-transfected with p14 plus empty vector, as shown by quantifying syncytial nuclei (Figure 1A, End construct) and from microscopic examination of transfected cells (Figure 1B). The p14 endodomain was capable of increasing the fusogenic activity of the full-length protein, but did not rescue the fusion-dead N-terminal (ΔEct) or C-terminal (ΔEnd) truncated versions of p14 (data not shown). The enhancing activity of the p14 endodomain was only significant at early times post-transfection (∼6–8 h for p14), and was not manifested by either ecto- or endodomain constructs that retained the p14 transmembrane domain (Figure 1A). Using the extent of syncytium formation in cells co-transfected with the p14 expression plasmid plus empty vector as a baseline, co-transfection of the non-fusogenic p14 endodomain with authentic p14 increased syncytiogenesis to 60–80% of that obtained in cells transfected with a double-dose of the full-length protein (Figure 1C). In other words, the non-fusogenic p14 endodomain functions almost as well as the full-length protein in enhancing p14-mediated syncytium formation. An N-terminal FLAG-tagged version of the p14 endodomain retained enhancement activity (Figure 1C), and Western blotting with an anti-FLAG antibody was used to confirm expression of the endodomain in transfected cells (Figure 1D). A scrambled version of the endodomain exhibited no enhancement capability (Figure 1C), suggesting this activity is sequence-specific.

**Figure 2:**
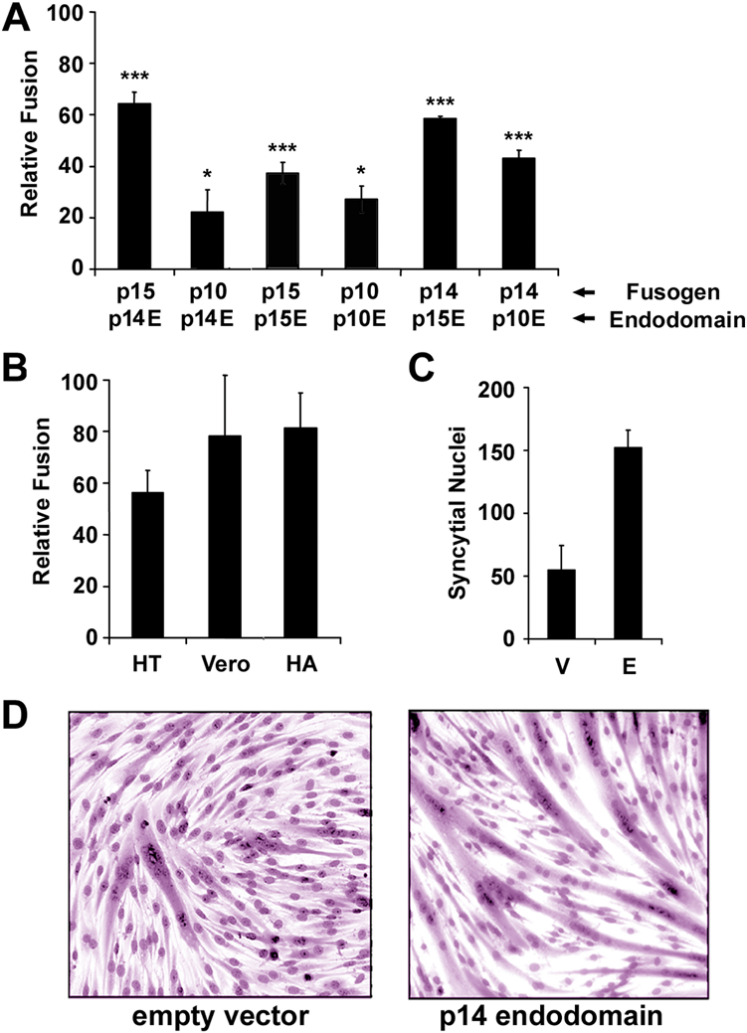
The FAST protein endodomains function as general enhancers of syncytium formation. (A) QM5 cells were co-transfected with plasmids expressing the indicated FAST proteins (NBV p10, p14, or p15) and the indicated endodomain (from p10 (p10E), p14 (p14E) or p15 (p15E)). The extent of cell–cell fusion was quantified as described in Figure 1A, and results are presented as the relative fusion level±S.E. (n = 4), setting the cells co-transfected with the full-length fusogen as 100% fusion enhancement and those co-transfected with empty vector as 0% fusion enhancement. Syncytia were quantified during the linear range of the fusion assay (12 h post-transfection for p10; 8 h post-transfection for p14 and p15). Statistically significant increases in syncytium formation relative to cells co-transfected with fusogen plus empty vector are indicated with asterisks (*p<0.05; ***p<0.001). (B) HT-1080 (HT) or Vero cells were co-transfected with plasmids expressing p14 and the p14 endodomain, or QM5 cells were co-transfected with the p14 endodomain and influenza virus hemagglutinin (HA). The relative fusion level±S.E. (n = 4) was determined as described in (A). (C) C2C12 murine myoblasts were transfected with the p14 endodomain expression plasmid or empty vector and induced to undergo differentiation-dependent cell–cell fusion. The extent of syncytium formation at 72 h post-transfection was quantified using a syncytial index, as described in Figure 1A, and results are reported as the average number of syncytial nuclei per field±S.E. (n = 4). (D) As for (C), with the extent of syncytium formation in C2C12 cells transfected with the p14 endodomain expression plasmid or empty vector visualized by bright field microscopy of Giemsa-stained monolayers at 72 h post-transfection.

To determine whether the bioactive property of the p14 endodomain was generally applicable to members of the FAST protein family, similar studies were conducted with the endodomains of the p10 and p15 FAST proteins, using both homotypic and heterotypic co-transfections. Since the kinetics of syncytium formation for the various FAST proteins varies widely [14], we determined the time range where doubling the dose of the fusogen yielded approximately twice the extent of syncytium formation. The enhancing activity of the endodomain fragments was quantified during this time range, which varied from 6–15 h post-transfection for the various FAST proteins. Results are presented as relative fusion, using cells transfected with a double-dose of the full-length fusogen as 100% fusion capacity and cells co-transfected with the fusogen plus empty vector as 0% fusion. The relative fusion scale accounts both for the varying times and the different extents of cell fusion mediated by the various FAST proteins (which ranged from ∼60–130 nuclei per field for single and double doses of p10, respectively, versus ∼390–770 syncytial nuclei per field for p14). Ectopic expression of the p10 and p15 endodomains enhanced syncytiogenesis mediated by their corresponding full-length FAST proteins, albeit at reduced levels compared to the p14 endodomain (Figure 2A), which could reflect either inherent differences in their enhancement activities or variable expression levels of the different endodomains. Interestingly, the activity of the various FAST protein endodomains was not confined to enhancing the activity of the corresponding full-length protein, since syncytium formation was consistently higher in cells co-transfected with various combinations of endodomain and FAST protein than in cells co-transfected with the fusogen plus empty vector (Figure 2A).

Using the more robust p14 endodomain as the prototype, we examined the cell-type and fusogen specificity of the endodomain enhancing activity. The syncytium-enhancing activity of the p14 endodomain was not cell-specific, functioning to approximately the same degree in human HT1080 fibroblast and monkey Vero epithelial cells as it did in QM5 quail fibroblasts (Figure 2B). Most interestingly, the p14 endodomain also enhanced the low pH-induced syncytium formation mediated by the unrelated influenza virus hemagglutinin (Figure 2B), and the syncytiogenic activity of the unidentified, endogenous fusogen(s) responsible for the differentiation-dependent fusion of C2C12 murine myoblasts into myotubes (Figure 2C and 2D). The 68-residue, non-membrane–anchored form of the p14 endodomain therefore has the surprising ability to function as a general enhancer of syncytiogenesis.

### The p14 Endodomain Enhances Syncytiogenesis, Not Membrane Fusion

**Figure 3:**
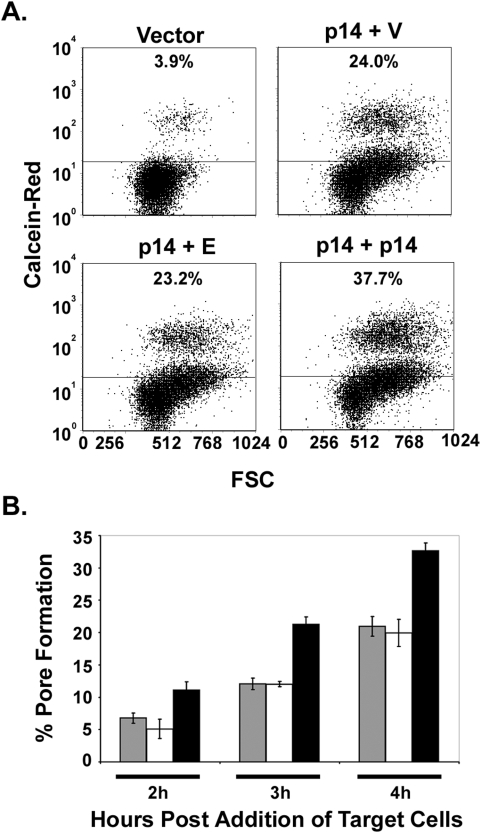
The p14 endodomain functions downstream of stable pore formation. (A) QM5 cells were co-transfected to express p14, EGFP, and either empty vector (p14+V), the p14 endodomain (p14+E), or full-length p14 (p14+p14), and 3 h post-transfection were over-seeded with Vero cells labelled with calcein red AM. The cells were co-cultured for 4 h to allow cell–cell fusion to proceed, then trypsinized, and single-cell suspensions were analyzed by flow cytometry. EGFP-expressing donor cells were gated, and the percent donor cells that acquired calcein red were quantified and plotted versus the forward scatter (FSC). Donor cells transfected with empty vector instead of p14 (Vector) served as a control for fusion-independent dye transfer. Data is representative of two experiments conducted in triplicate. (B) A time course analysis of the experiment described in (A). The percent donor cells positive for calcein red, minus the background from vector-transfected donor cells, is graphed as percent pore formation. Results are the mean±S.D. from a representative experiment in triplicate. Cells were transfected with p14+vector (grey), p14+endodomain (white), or a double-dose of p14 (black).

A cell–cell pore-forming assay was used to determine whether the p14 endodomain peptide enhanced syncytiogenesis prior or subsequent to the formation of stable fusion pores. QM5 fibroblasts co-expressing p14, EGFP and either empty vector or the p14 endodomain plasmid were co-cultured with Vero cells labelled with the small aqueous fluor calcein red-orange. The extent of fusion pore formation was estimated using flow cytometry to quantify the percent of EGFP-containing donor cells that acquired calcein red from the target cells. Cells transfected with vector alone displayed a low level of spontaneous dye transfer while expression of p14 resulted in a time-dependent increase in the percent of co-fluorescent cells that coincided with the appearance of syncytia (Figure 3). In independent experiments, doubling the dose of p14 resulted in a 1.6–2.2 fold increase in pore formation (depending on the time point), but unlike the syncytiogenesis assay, pore formation in cells co-expressing p14 and the endodomain peptide was indistinguishable from cells co-expressing p14 and empty vector (Figure 3A). In duplicate experiments conducted in triplicate, examining multiple time points over the linear time course of the pore formation assay (Figure 3B), the extent of pore formation in cells expressing p14 plus the endodomain never exceeded that observed in control cells expressing p14 plus empty vector. The p14 endodomain therefore has no inherent ability on its own to promote pore formation or syncytiogenesis, but it displays the remarkable ability to enhance the syncytiogenic activity of functional p14, and this enhancing activity exerts its effect subsequent to the formation of stable fusion pores.

### Endogenous *In Vivo* Generation of the p14 Endodomain

**Figure 4:**
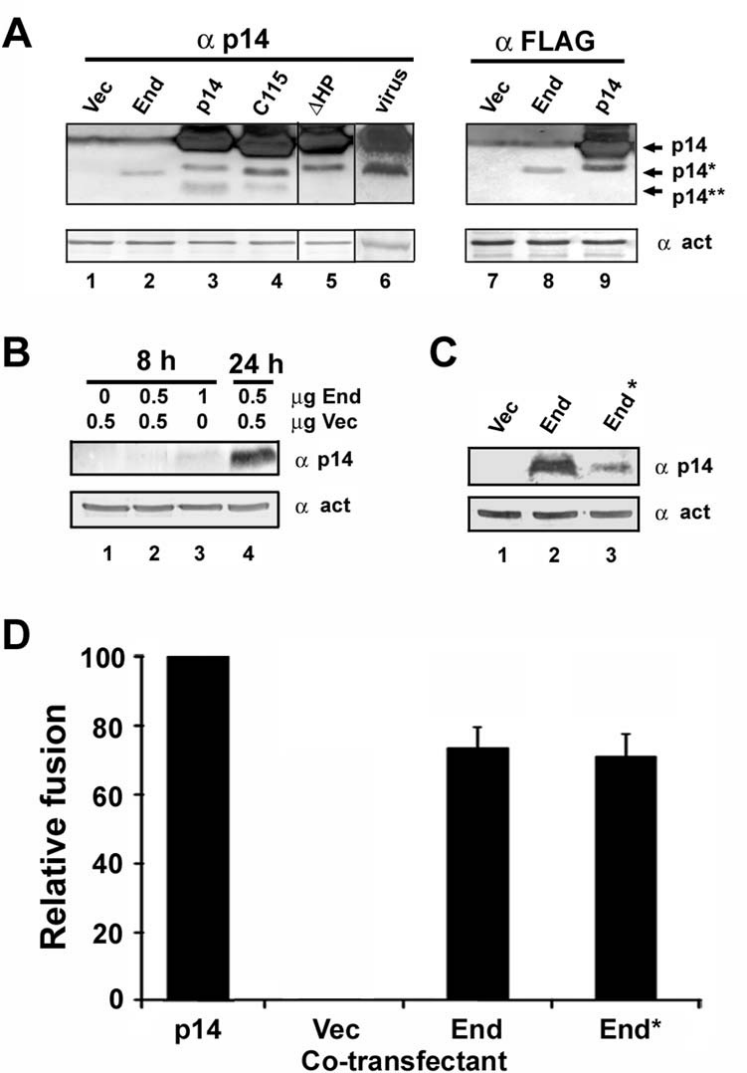
Endogenous *in vivo* generation of the p14 endodomain. (A) Cell lysates from QM5 cells infected with reptilian reovirus (lane 6), or transfected with empty vector (lanes 1 and 7) or with plasmids expressing the p14 endodomain (End, lane 2), full length p14 (p14, lane 3), a C-terminal 10-residue (C115, lane 4) or N-terminal 21-residue (ΔHP, lane 5) truncation of p14, or N- (lane 8) or C- (lane 9) terminally FLAG-tagged versions of the endodomain or p14 were processed for Western blotting at 20 h post-infection or 12 h post-transfection using antibodies against p14, the FLAG epitope, or β-actin. The migration of full-length p14, the p14 endodomain (p14*), and the presumed p14 ectodomain (p14**) fragments are indicated on the right. Lane 5 was spliced in from the same blot as lanes 1–4; lane 6 was spliced in from a separate blot. (B) QM5 cells were transfected with the indicated amounts of empty vector (Vec) or with the p14 endodomain expression plasmid (End). At 8 or 24 h post-transfection, cell lysates were harvested and processed for Western blotting using antibodies against p14 or β-actin. (C) QM5 cells were transfected with the p14 endodomain expression plasmid (End) or with a plasmid expressing p14 from a sub-optimal translation start codon (End*). Cell lysates were harvested at 24 h post-transfection and processed for Western blotting using antibodies against p14 or β-actin. (D) Cells transfected with 0.5 µg of the p14 expression plasmid were co-transfected with 0.5 µg of plasmids expressing authentic p14 (p14), empty vector (Vec), the p14 endodomain (End), or expressing p14 from a sub-optimal translation start codon (End*). The extent of cell–cell fusion in triplicate samples was quantified as described in Figure 1A, and results are presented as the relative level of syncytium formation±S.D. from a single experiment, setting the cells co-transfected with authentic p14 as 100% fusion enhancement and those co-transfected with empty vector as 0% fusion enhancement.

To determine whether the endodomain fragment is naturally generated in cells transfected with only the full-length p14 protein, Western blots of p14-transfected cell lysates obtained 12 h post-transfection were probed using a polyclonal antiserum raised against the p14 protein. In addition to full-length p14, these blots clearly detected sub-molar amounts of a p14 fragment whose gel mobility closely approximated that of the ectopically expressed p14 endodomain (Figure 4; p14*). In addition, a second, smaller p14 fragment was detected on some blots (Figure 4; p14**), but at reduced levels relative to the p14* fragment. Neither of these fragments (p14* and p14**) was ever detected in lysates from vector-transfected cells (Figure 4, lane 1). A ten-residue C-terminal truncation of p14 increased the gel mobility of both the p14 and p14* polypeptides but not the p14** fragment (Figure 4, lane 4), while a 21-residue N-terminal truncation eliminated detection of the p14** fragment with no effect on mobility of the p14* polypeptide (Figure 4, lane 5). These results suggested proteolytic processing of the full-length p14 protein generated the p14* endodomain fragment and the corresponding p14** N-terminal fragment, which was either further degraded or shed from membranes resulting in reduced or undetectable steady state levels of this fragment. Confirmation that p14* represented endogenous generation of the p14 endodomain was obtained using a p14 construct containing a C-terminal FLAG tag. Western blot analysis using an anti-FLAG antibody detected both the p14 and p14* polypeptides but never the p14** fragment (Figure 4, lane 9). Most notably, a fragment representing the p14 endodomain was also detected in Vero cells infected with reptilian reovirus (Figure 4, lane 6), and the levels of the p14 endodomains endogenously generated in transfected or virus-infected cells were equivalent to, or exceeded, those observed by ectopic expression. The biological activity displayed by ectopic expression of the p14 endodomain is therefore not due to artificial over-expression of the peptide, and the same endodomain fragment is endogenously generated by proteolytic processing of a percentage of the p14 protein at concentrations sufficient to serve as an enhancer of syncytiogenesis.

Since the p14 endodomain is endogenously generated from the full-length protein at levels equivalent to those obtained by exogenous expression and sufficient to be bioactive, this raised the question as to the relative contribution of the endogenous and exogenous endodomains to syncytiogenesis. The endogenous and exogenous endodomains were both detectible at similar levels 12 h post-transfection (Figure 4A), ∼4 h after the time when the exogenous endodomain exerts a significant enhancing effect on syncytiogenesis. Expression levels of the endogenous (data not shown) and exogenous (Figure 4B) endodomains were below detectible levels by Western blotting at 6–8 h post-transfection, when syncytial enhancement was evident. Doubling the dose of the ectopic endodomain resulted in barely detectible levels by 8 h post-transfection (Figure 4B, lane 3). These results suggested that low levels of the endodomain are sufficient to exert an enhancing effect on syncytium formation. This conclusion was further supported by converting the optimized Kozak consensus sequence used for translation initiation of the exogenous endodomain (ACCAUGG) to a sub-optimal sequence (CTTAUGA) [28]. This change in the translation start site substantially reduced expression levels of the exogenous endodomain, as shown at 24 h post-transfection to reveal the low level of expression from the sub-optimal translation start site (Figure 4C), but had no significant effect on diminishing fusion enhancement activity (Figure 4D). The p14 endodomain therefore displays bioactive properties at low levels of intracellular expression. However, since only a small percentage of p14 is processed to generate the endodomain, it seems likely that the endogenous endodomain will exist at sub-saturating levels at early times post-transfection, which may explain why ectopic expression enhanced syncytiogenesis at early times but not at later times when the endogenous endodomain may reach saturating levels.

### The Endodomain Functions as a Soluble Syncytiogenic Enhancer

**Figure 5:**
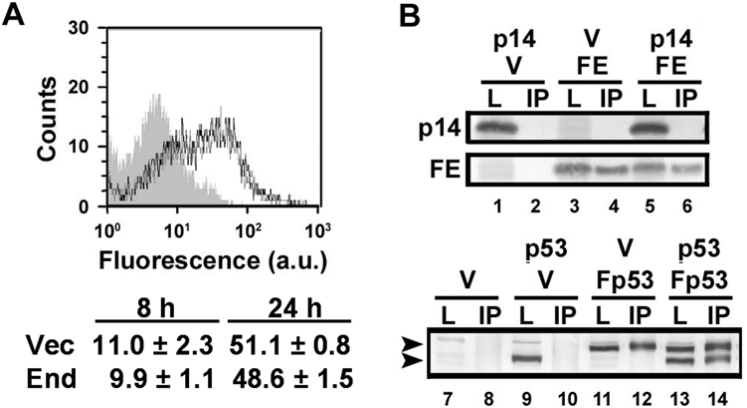
The endodomain functions as a fusion enhancer independent of direct interactions with the fusogen. (A) Top panel: Live cells co-transfected with p14G2A and the p14 endodomain (black lines) or empty vector (grey lines) were immunostained using anti-p14ecto antiserum and analysed by flow cytometry. Grey-filled histogram represents auto-fluorescence from mock-transfected cells. Bottom panel: Surface expression of p14G2A at 8 h and 24 h post-transfection in cells co-transfected with empty vector (Vec) or the p14 endodomain (End), as determined by flow cytometry and Overton subtraction relative to mock-transfected cells. Numbers indicate the mean fluorescence intensity increase above mock-transfected cells±S.D. from a representative experiment in triplicate. (B) Cells were co-transfected with the indicated expression plasmids (p14, authentic p14; V, empty vector; FE, FLAG-tagged endodomain; p53, authentic p53; Fp53, FLAG-tagged p53), and cell lysates were immunoprecipitated using anti-FLAG antibody. The immunoprecipitates (IP) and unfractionated cell lysates (L) were processed for Western blotting using anti-p14 (top panel) or anti-p53 (bottom panel) antibodies. The top arrow in the bottom panel indicates the migration of FLAG-tagged p53 and the bottom arrow untagged p53.

A biological and biophysical characterization of the endodomain was undertaken to gain some insight into how this peptide fragment might exert its enhancing activity. Co-expression analysis indicated the endodomain did not increase the steady-state levels of p14 (see Figure 1D). To determine whether the p14 endodomain altered cell surface expression of p14, cells were co-transfected with the p14 endodomain and p14G2A, a fusion-minus mutant of p14 that displays normal cell surface expression [11] (p14G2A avoided the complications associated with analyzing large syncytia by flow cytometry). Live cells were immunostained using an antiserum specific for the p14 ectodomain. As indicated (Figure 5A), the endodomain did not enhance syncytiogenesis by increasing the surface expression of p14. The ability of the p14 endodomain to enhance syncytiogenesis mediated by heterologous fusogens makes direct physical interactions between the endodomain and the fusogen unlikely. This was further confirmed by immunoprecipitation of the FLAG-tagged endodomain construct using anti-FLAG antibody, which did not result in co-precipitation of the full-length p14 protein (Figure 5B). Similar analysis of a known multimeric protein, p53, provided a positive control for the co-immunoprecipitation assay (Figure 5B). The p14 endodomain therefore does not exert its biologically activity via direct interactions with the fusogen.

**Figure 6:**
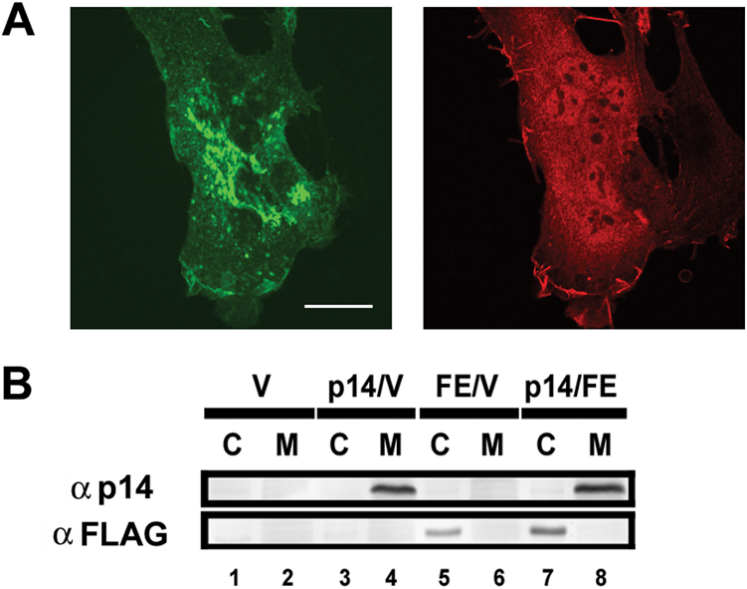
The p14 endodomain is a soluble nucleocytoplasmic peptide. (A) Cells were co-transfected with HA-tagged p14 and FLAG-tagged p14 endodomain, fixed and permeabilized at 8 h post-transfection, and stained using rabbit anti-HA and mouse anti-FLAG antibodies and appropriate fluorescently tagged secondary antibodies. The HA-tagged p14 (left panel) exhibited punctate, reticular staining in the cytoplasm while the FLAG-tagged p14 endodomain (right panel) was broadly distributed throughout the cytosol and nucleus. Scale bar = 20 µm. (B) Cells were co-transfected with the indicated expression plasmids (p14, full-length p14; V, empty vector; FE, FLAG-tagged p14 endodomain), and cell lysates were fractionated into the cytosolic “C” and membrane “M” fractions before being processed for Western blotting using anti-p14 or anti-FLAG antibodies (indicated on the left).

Analysis of the subcellular distribution of the p14 endodomain by immunofluorescence microscopy revealed a diffuse cytosolic/nuclear staining pattern (Figure 6A). In contrast, and as previously reported [11], the p14 protein displayed the reticular and surface staining pattern characteristic of an integral membrane protein. Subcellular fractionation further indicated the endodomain is a soluble polypeptide, residing within the cytosolic fraction while p14 is found exclusively in the membrane fraction of cells (Figure 6B). Coupled with the observation that the membrane-anchored version of the endodomain did not augment p14-induced cell–cell fusion (ΔEct in Figure 1A), these results imply the endodomain exerts its enhancement activity independent of direct interactions with the membranes being fused.

**Figure 7:**
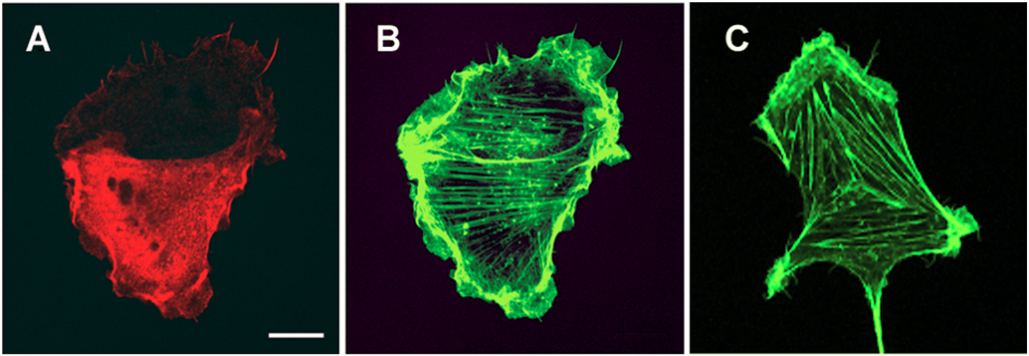
The actin cytoskeleton is unaffected by the p14 endodomain. QM5 cells were transfected with the FLAG-tagged p14 endodomain, and immunostained using anti-FLAG antibody and fluorescently tagged secondary antibody (A). F-actin was visualized by staining with FITC-phalloidin (B,C). A transfected cell expressing the FLAG-tagged p14 endodomain and an adjoining cell either not transfected or expressing undetectable levels of the endodomain ((A), lower and upper cells, respectively) exhibited similar arrangements of F-actin, as detected using FITC-phalloidin (B), and the same actin organization was observed in mock-transfected cells (C). Scale bar = 10 µm.

The ability of the endodomain to serve as a general enhancer of syncytiogenesis, functioning in *trans* from a separate subcellular location as the fusogen, suggested the endodomain influences an intracellular process common to all cell–cell fusion reactions. In view of the generic role of dynamic actin remodelling on membrane fusion events [29], we examined whether ectopic expression of the p14 endodomain resulted in cytoskeletal rearrangements. Staining F-actin in transfected and non-transfected cells using fluorescent phalloidin revealed no observable differences in the overall architecture of the actin cytoskeleton (Figure 7), suggesting that any effects of the endodomain on actin are not manifested by gross changes in the structure of the cytoskeleton. This does not preclude the possibility that more subtle effects of the endodomain on actin distribution might influence its *trans*-enhancing activity.

### Multiple Regions of the p14 Endodomain Influence Enhancement Activity

**Figure 8:**
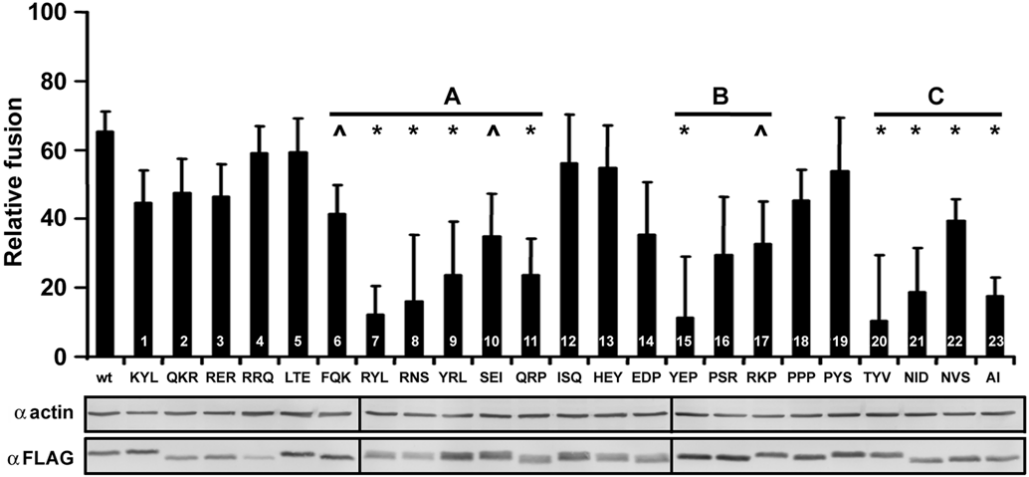
Multiple regions of the p14 endodomain affect *trans*-enhancing activity. Every amino acid of the p14 endodomain, in groups of three consecutive residues (indicated along the x-axis), was substituted with alanine. These endodomain alanine mutants (numbered 1–23) were co-expressed with authentic p14 in co-transfected QM5 cells, and the relative fusion level±S.E. (n = 4) was determined as described in Figure 1C. Three regions, “A”, “B”, “C”, where alanine substitutions resulted in a more pronounced inhibitory effect on the *trans*-potentiation activity of the endodomain, are indicated on the graph. p14 constructs displaying a significant decrease (p<0.05) in fusion enhancement activity (*), and those approaching (p<0.06) statistical significance (∧), are indicated. Western blots with anti-FLAG antibodies were used to assess expression levels of the various FLAG-tagged endodomain mutants, using actin blots as a loading control.

The enhancing activity of the p14 endodomain is sequence-specific, as indicated by the inability of a scrambled endodomain construct to enhance cell–cell fusion (see Figure 1C), suggesting a linear motif may be important in the enhancement mechanism. The p14 endodomain contains a membrane-proximal polybasic region (KRRERRR) and a C-proximal polyproline region (PYEPPSRRKPPPPP) that contains a pentaproline motif and a PXXP motif, a ligand for SH3 domains [30]. To determine whether these, or other, motifs might contribute to endodomain fusion enhancement activity, we conducted an alanine scan, substituting consecutive groups of three amino acids with alanine residues. These 23 endodomain mutants were quantitatively assessed for their enhancing capacity (Figure 8). Western blot analysis of the FLAG-tagged mutants revealed slight variations in steady-state levels, but well within the range of expression levels previously shown to be saturating for enhancement activity (see Figure 4C). The three alanine mutants spanning the polybasic region (Figure 8, bars 2–4) had little if any deleterious effect on the capacity of the endodomain to enhance cell–cell fusion, implying the polybasic region does not exert a significant effect on the fusion enhancing activity of the endodomain. Three other regions of the endodomain were, however, sensitive to alanine substitutions. Region A lies between the polybasic and polyproline motifs, and several substitutions in this region had adverse effects on fusion enhancement (Figure 8, bars 7–11). These substitutions affect two potential protein kinase A recognition sites (XRX[ST}XXX), identified using the Eukaryotic Linear Motifs resource (ELM; http://elm.eu.org). Region B (Figure 8, bars 15–17) occurs in the endodomain polyproline region; alanine substitutions in this region that affected the pentaproline motif (Figure 8, bars 17–19) had no significant effect on enhancement activity while disruption of the PXXP motif (PAAA; Figure 8, bar 15) severely restricted enhancement activity. However, the PAAA substitution affects not only the PXXP motif, but also a predicted src homology-2 (SH2) ligand motif (YEPP). Mutant 14, which eliminated the PXXP motif but not the YEPP SH2 domain-binding motif, was not significantly impaired in its enhanced syncytiogenic activity (Figure 8), suggesting the potential SH3 domain PXXP ligand motif is unlikely to contribute to the enhancing activity of the endodomain fragment. All four of the substitution mutants contained within region C, the extreme C-terminus of the endodomain, displayed significantly diminished enhancing activity. This C-terminal region includes potential SH2 (Y[IV]X[VILP]) and PDZ (X[DE]X[IVL] or X[ST]X[VIL]) ligand motifs. Therefore, several regions of the 68-residue p14 ectodomain contain potential linear motifs or structural determinants involved in the ability of this soluble peptide fragment to function as a general enhancer of syncytiogenesis.

## Discussion

The reovirus FAST proteins are a new family of viral fusogens whose structural and functional properties differ extensively from the well-characterized fusion proteins encoded by the enveloped viruses. The unusual topology of the FAST proteins positions ∼60–90% of their mass within the transmembrane and endodomains, suggesting the mechanism by which they induce cell–cell fusion and syncytium formation is particularly focused on the donor cell, the membrane in which they reside. We recently demonstrated that the FAST proteins rely on surrogate cellular adhesins to mediate the membrane attachment and close apposition stages of the fusion reaction [31]. This observation provided the first explanation for the exceedingly small size of the FAST protein ectodomains, which are charged with promoting the fusion of closely apposed lipid bilayers, not with bringing the membranes into close proximity. We now show that an additional explanation for the unusual asymmetric membrane topology of the FAST proteins reflects the generation of a soluble endodomain fragment which functions as a general enhancer of syncytium formation, functioning in *trans* to promote the conversion of fusion pores into syncytia. The use of surrogate adhesins coupled with the generation of a bioactive endodomain peptide presumably reflects the unique evolution of the FAST proteins as virus-encoded cell–cell fusogens, allowing these diminutive cell–cell fusogens to efficiently induce syncytium formation within the confines of their rudimentary structures.

Since the endodomain fragment is endogenously generated from full-length p14, both in transfected and virus-infected cells (Figure 4), it seems likely that the enhancing activity of the endodomain is relevant to the mechanism of p14-induced syncytium formation. Additional observations support this speculation. C-terminal residues influence the enhancing, though non-essential, syncytiogenic activity of the soluble endodomain (Figure 8). In the context of the full-length protein, C-terminal truncation of p14, which generates a truncated endodomain fragment (Figure 4), simultaneously reduces the rate, but not the final extent, of p14-induced syncytiogenesis by ∼50% [11]. The C-terminus of the full-length p14 protein therefore enhances syncytiogenic activity, and this same region is essential for the *trans*-acting activity of the soluble endodomain. Results further indicate that low steady state levels of the endodomain are all that is required for biological activity (Figure 4). The sensitivity of Western blots was not sufficient to correlate fusion enhancement activity with the steady state levels of the exogenous and endogenous endodomains at early time points. Nonetheless, intracellular concentrations of the endogenously generated endodomain exceed bioactive levels at slightly later time points, consistent with the concept that the enhancing activity of the soluble endodomain is relevant to the natural function of p14 as a cell–cell fusogen. The expression data also serves to explain why ectopic endodomain expression would augment the enhancement activity of the endogenous soluble endodomain, functioning at early times post-transfection to increase the rate at which the soluble endodomain accumulates to bioactive levels inside cells.

In addition to the C-terminus, other regions of the endodomain that affect its enhancing activity contain potential protein–protein interaction motifs (Figure 8). The degenerate nature of the consensus sequences for these motifs makes it unclear whether the endodomain deletion and substitution results reflect disruption of a specific linear motif or global changes to the endodomain structure. If specific linear motifs do contribute to endodomain function, then predicted protein kinase A sites and SH2 and PDZ domain-binding motifs present in the p14 endodomain may be involved. These motifs are widely involved in diverse cell signalling pathways that could influence the efficiency by which the cell promotes the conversion of fusion pores to syncytia [32]–[35]. Since all of the FAST protein endodomains appear to contain at least some level of *trans*-enhancing activity (Figure 2A), it seems reasonable to assume they might function through the same cellular pathway. It also seems reasonable to assume that the potential protei–protein interactions motifs identified in the p14 endodomain alanine scan might be conserved in the FAST protein endodomains, even though the FAST protein endodomains lack any extended regions of direct sequence conservation. An ELM scan of the p10 and p15 endodomains identified numerous potential protein interaction or post-translational modification motifs. The only common motifs identified in all three endodomains were different classes of PDZ domain ligands, which occur at the C-terminus of p14 and p10, but internally in the p15 endodomain. Whether these motifs are relevant to the bioactive property of the endodomain and if so, how mutations outside these motifs influence their role in protein interactions remains to be determined. NMR structural analyses of the FAST protein endodomains coupled with pull-down assays are currently underway to assist in interpretation of the mutagenic analyses and to identify cellular partners that may serve as effectors of endodomain bioactivity.

There are no direct parallels in the viral membrane fusion protein field to the *trans*-acting enhancement activity of the p14 endodomain. There are examples where enveloped viral fusion proteins are proteolytically cleaved, for example the maturation cleavage involved in the assembly stage of several retroviruses [36],[37]. In this instance, cleavage activates the fusion complex by removal of an inhibitory C-terminal peptide, rather than by generating a functional peptide fragment. Similar to the *trans*-enhancing activity of the soluble p14 endodomain, an artificially truncated version of the fusogenic vesicular stomatitis virus G protein comprised of the endodomain, transmembrane domain and a fragment of the ectodomain enhances the fusion activity of heterologous fusogens [38]. However, this membrane-anchored G-stem polypeptide appears to influence the membrane apposition and/or hemifusion stages of the fusion reaction, which is clearly not the case with the soluble p14 endodomain peptide that functions in an indirect manner, independent of direct membrane interactions, to promote fusion pore expansion. In *C. elegans*, the Eff-1 fusogen involved in developmental epithelial cell–cell fusion generates a soluble ectodomain fragment that enhances syncytiogenesis [3],[9]. This fragment has no demonstrated role in enhancing the activity of heterologous fusogens, and it seems unlikely that it would function from the extracellular milieu in a similar manner as the cytosolic p14 endodomain. The features of the *trans*-acting activity of the p14 endodomain are therefore unique amongst both viral and cellular fusogens.

While the precise mechanism by which the soluble FAST protein endodomains enhance syncytiogenesis remains to be determined, several features of this mechanism are apparent. Coupled with observations from other studies, these results provide some interesting into insights into this remarkable biological activity. The ability of the endodomain to enhance syncytiogenesis mediated by the influenza HA fusogen (which occurs within minutes after triggering by treatment with low pH), and the gradual cell–cell fusion induced by the FAST proteins and the endogenous fusogens responsible for myoblast fusion, which induce fusion over hours or days, suggests the effects of the endodomain are constant and sustained over time. Expression of the endodomain did not alter overall cell function since cell morphology and growth properties were not affected (Figure 7), suggesting the p14 endodomain likely functions in a somewhat specific manner. Furthermore, low steady state levels of the endodomain are all that is required to enhance a step in syncytium formation that occurs after formation of stable fusion pores (Figures 3 and 4) in a manner that is not dependent on direct physical interactions with either the fusogen or the membranes being fused (Figures 5 and 6). Taken together, the most straightforward explanation for the ability of the p14 endodomain to function as a general enhancer of syncytiogenesis is that the endodomain functions as a signalling peptide to activate or recruit an intracellular pathway broadly involved in the conversion of cell–cell fusion pores to syncytia.

We know of no system where the mechanism by which fusion pores expand into syncytia has been clearly defined. In *C. elegans*, epithelial cell fusion has been kinetically divided into two distinct stages designated microfusion, the actual membrane fusion event that results in rapid and stable pore formation, and macrofusion, a slower pore expansion stage required for syncytium formation [9],[10]. A similar, kinetically distinct two-stage process has been demonstrated to occur during yeast mating, where fusion pores (i.e. microfusion) open quickly and reversibly, followed by slow expansion and macrofusion [39]. Various explanations for how fusion pores might expand to the macrofusion stage have been put forward. These scenarios include, but are not limited to, membrane removal by vesiculation [10], lateral membrane tension [39], direct or indirect effects of the fusion protein itself [7],[25], and actin-driven effects on membrane tension [29],[40]. There is also evidence that the rate of pore expansion is influenced by the cell type [25], suggesting there are cellular pathways that directly influence the macrofusion stage of syncytiogenesis. We therefore propose that the soluble endodomains of the FAST proteins harness a cellular pathway involved in driving the transition from microfusion to macrofusion, perhaps the most energy demanding stage of syncytiogenesis [2],[23].

There are interesting parallels between the ability of the FAST proteins to generate a bioactive, soluble endodomain peptide, and membrane receptors and ligands that undergo regulated intramembrane proteolysis (RIP) [41],[42]. Proteins such as sterol-regulatory-element–binding protein (SREBP) and the Notch receptor are two well-characterized examples of membrane protein substrates that undergo RIP to mediate membrane-to-nucleus signalling. Cleavage by intramembrane cleaving proteases (iCLIPs), such as the presenilin/γ-secretase complex or the site-2 protease, results in release of a bioactive cytoplasmic domain that translocates to the nucleus to initiate signalling cascades that regulate lipid metabolism or diverse cell differentiation processes [43]–[45]. We note that the endogenously generated p14 endodomain fragments were consistently slightly larger than the ectopically expressed endodomain (Figure 4), suggesting that p14 may also be processed within its transmembrane domain by iCLIPs to generate the bioactive endodomain peptide. Since the soluble p14 endodomain exists as a nucleocytoplasmic peptide (Figure 6), interaction with cellular proteins in either compartment could alter cellular signalling pathways important in the process that drives expansion of cell–cell fusion pores. Although the soluble endodomain clearly has *trans*-acting activity, only a small percent of p14 is processed to generate the soluble endodomain. It therefore seems likely that the endodomains of the FAST proteins may also function in *cis* to influence cell–cell fusion activity. A similar dual *cis/trans* function has been proposed for other type I membrane proteins that undergo RIP, for instance the Notch receptor ligand Jagged-1 that interacts in *cis* with proteins involved in organizing cell–cell junctions while functioning in *trans* as a signalling peptide [46],[47].

The FAST proteins are the first example of a fusion protein that naturally generates a *trans*-acting subunit capable of modulating a cellular pathway or process that may be common to all biological cell–cell fusion events. By promoting the transition of fusion pores into syncytia, the *trans*-acting activity of the C-terminal tail of the FAST proteins allows these simple cell–cell fusogens to efficiently induce syncytium formation within the confines of their rudimentary structures. Clearly, numerous questions regarding the function of the FAST protein endodomains remain to be addressed. What, if any, *cis*-acting role is exerted by the endodomain? What regulates p14 processing and why is only a small percentage cleaved? Does the soluble endodomain exert its enhancing activity from the cytoplasm and/or nucleus? What cellular partners interact with the soluble endodomain, what pathways are regulated by these partners, and how do these pathways promote fusion pore expansion and syncytium formation? Most importantly, the general enhancing activity of the p14 endodomain suggests that discovering the effectors regulated by the p14 endodomain may provide insights into cellular pathways that are central to the process of cell–cell fusion in a diversity of biological processes.

## Materials and Methods

### Clones

The cDNA clones of the NBV p10, p14, and p15 FAST proteins in pcDNA3 (Invitrogen) were previously described [11]–[13]. Standard PCR techniques were used to generate the p10 and p15 endodomain constructs, and the p14 endodomain (End, residues 58–125), scrambled p14 endodomain (SEnd), N- (ΔEct, residues 35–125) and C- (ΔEnd, residues 1–78) terminally truncated p14, and N- (EΔN, residues 35–125) and C- (EΔC, residues 1–78) terminally truncated p14 endodomain expression plasmids. Each N-terminal truncation included an additional alanine residue immediately following the initiator methionine, a consequence of optimizing the context of the translation start site [13]. The p14 endodomain was subjected to alanine scan mutagenesis, substituting consecutive groups of three amino acids with alanine residues, using nested primers and standard PCR techniques. All constructs were confirmed by sequencing. The influenza hemagglutinin (strain X-31) was a gift from Judy White, and was subcloned into pcDNA3. The N-terminal 3× FLAG-tagged p14 endodomain (F-End) construct was obtained by subcloning into pBICEP (Sigma).

### Cells and Reagents

QM5 and Vero cells were grown and maintained as previously described [11]. HT1080 and C2C12 cells were cultured in MEM or DMEM, respectively, supplemented with penicillin/streptomycin (50 µg/ml) and 10% FBS. The C2C12 myoblasts were induced to differentiate into myotubes by culturing the cells in DMEM supplemented with 2% horse serum for 72 h. The rabbit antiserum against full-length p14 was previously described [11]. Rabbit antiserum against the p14 ectodomain (residues 1–36) was prepared by New England Peptide (anti-p14ecto). Mouse anti-FLAG antibodies (Sigma), horseradish peroxidase-conjugated goat anti-rabbit (KPL) and goat anti-mouse (Jackson ImmunoResearch) secondary antibodies, Alexa-488-conjugated goat anti-rabbit IgG and Alexa-555-conjugated goat anti-mouse IgG secondary antibodies (Invitrogen) were from the indicated commercial sources. FITC-conjugated phalloidin was from Molecular Probes.

### Transfection and Syncytial Index

Cells at 70–80% confluency in 12-well cluster plates were transfected or co-transfected with equivalent quantities (0.5 µg) of the various expression plasmids using Lipofectamine (Invitrogen), then supplemented with appropriate serum-containing medium 5 h post-transfection. Transfected cells were fixed at different times post-transfection based on control experiments that determined the linear dose-response range (i.e., cells transfected with 1 µg of the p14 expression plasmid yielded twice the level of fusion as cells transfected with 0.5 µg of the same plasmid). Cells expressing HA were trypsin-activated and fusion was induced by low pH treatment as previously described [31]. A syncytial index from triplicate samples was determined as previously described [14], by microscopic examination to quantify the average number of syncytial nuclei per field from five random fields of the Giemsa-stained monolayers. The syncytial index was converted to a relative fusion scale to permit comparisons between replicate experiments (n>3) using the formula (C_fe_−C_fv_/C_ff_−C_eV_)×100. This formula sets cells co-transfected with the fusogen plus empty vector (C_fv_) as the baseline and cells transfected with a double-dose of the fusogen (C_ff_) as the maximum possible extent of fusion (100%), and quantifies the extent to which cells co-transfected with the fusogen plus the p14 endodomain (C_fe_) approach the fusion maximum. Results were analyzed using a two-tailed unlinked t-test to determine statistical significance.

### Western Blotting

QM5 cells were lysed in RIPA buffer (50 mM Tris, pH 8.0, 150 mM NaCl, 1 mM EDTA, 1% Igepal, 0.5% SDS) at 8–24 h post-transfection and equivalent protein loads were analyzed by Western blotting, as previously described [13]. Cell lysates were similarly prepared from cells infected with reptilian reovirus [48] for 20 h. For detection of the sub-molar, endogenously generated endodomain fragment, the anti-p14 antiserum was used at 1∶3000 dilution instead of 1∶10,000.

### FACS-Based Fusion Assay

Sub-confluent monolayers of QM5 fibroblasts were co-transfected with plasmids expressing p14 and EGFP and either empty vector or the p14 endodomain plasmid. At 4 h post-transfection, these cells were overlaid with Vero cells (5∶1 ratio of Vero to QM5) labelled with 20 µM calcein red-orange AM (Molecular Probes). The two cell populations were co-cultured at 37°C to allow fusion to proceed. At various times (2–4 h), the cell cultures were detached from the substratum, fixed and analyzed by flow cytometry (FACSCalibur (Becton Dickinson)) using appropriate filter sets and Cell Quest software. EGFP-positive donor cells were gated, and the percent of these donor cells that acquired calcein red was quantified. A minimum of 10,000 events were recorded, and all data were analyzed using FSC Express 2.0 (De Novo Software).

### Cell Surface Expression

Cells were co-transfected with p14G2A, a fusion-minus mutant of p14 that displays normal cell surface expression (this mutant was used to avoid the complication of trying to analyze large syncytia by flow cytometry) and either empty vector or the p14 endodomain. Transfected cells were washed with PBS supplemented with 5% BSA at 8–24 h post-transfection, and cells were then incubated with 1∶200 dilution of anti-p14ecto antiserum followed by 1∶2000 dilution of Alexa-647–conjugated goat anti-rabbit antibody. Cells were detached from the substratum with 50 mM EDTA in PBS and analysed by flow cytometry.

### Fluorescent Immunomicroscopy

Transfected cells grown on gelatin-coated coverslips were fixed at various times post-transfection using 3.7% formaldehyde, and permeabilized with 0.1% Triton X-100. The cells were blocked with normal goat serum, then stained using rabbit anti-HA and mouse anti-FLAG antibodies (1∶200 and 1∶2000, respectively) and Alexa-488–conjugated goat anti-rabbit IgG and Alexa-555–conjugated goat anti-mouse IgG (Invitrogen). Images were captured using a Zeiss META 510 confocal microscope.
